# Pathology and Treatment of Syphilis

**Published:** 1886-12

**Authors:** 


PATHOLOGY AND TREATMENT OF SYPHILIS. By H. Von Zeissl, M. D., late Professor at the University of Vienna. Second edition revised by M. Von Zeissl, M. D. Privat-Docent for the diseases of the skin and syphilis, University of Vienna. Translated by H. Raphael, M. D. D. Appleton & Co., 1886. 8vo., pp. 402.

This book is divided into three sections. Section I. on gonorrhœa gives a description of the nature and treatment of this disease, together with its complications and sequelæ, in both the male and female. Section II. discusses chancroid, its pathology and treatment. Section III., occupying two-thirds of the book, is on the subject of syphilis. The nature of the virus and characteristics of the initial lesion are first discussed, then follows a succinct description of all the constitutional manifestations, such as the cutaneous eruptions, lesions of the mucous membrane and the different internal organs, affections of the brain, spinal cord, eye, bones, muscles, joints and finally the subject of hereditary syphilis. Then is presented the subject of the treatment of syphilis, and here are found views that are at variance with those generally accepted in this country. The rationale of the various methods and their respective advantages are also given. Of late the subject of excision of the initial lesion to abort syphilis has acquired a fresh interest through the labors of Auspitz and Unna. These authorities declared that excision was an efficient prophylactic measure. Zeissl, also basing his conclusions upon experiment, reports against this view. In the treatment of syphilis specific remedies are withheld, even after the appearance of cutaneous manifestations, if the latter are in the form of a macular papular eruption. If the symptoms do not disappear in eight weeks the iodides are given, and if the symptoms continue after another period of eight weeks mercury is then resorted to. It is claimed that the early use of mercury is more apt to be followed by relapses than if it is used later, and that it should be used then only according to the severity of the symptoms. The inunction method is preferred. On page 344 he says:

"Iodines, (iodides) in proper quantities, in conjunction with a carefully regulated regimen, are sufficient to cause the symptoms of syphilis to disappear, or at least to be weakened so that only a few mercurial inunctions will be necessary to complete the cure without fear of a relapse occurring in years to come." This differs greatly from the ideas of treatment gained from the English text-books, but coming from the authority it does, it should command consideration. In the study of syphilis it requires an abundance of clinical material, and to judge of the comparative efficacy of methods of treatment it is necessary to keep patients under observation for years. Zeissl has had the material for study and has devoted a life-time to its elaboration. A careful examination of this work gives evidence of a rich personal experience and laborious research. The style is pleasing and the numerous subdivisions of the subject make the book easy for reference. It is replete with formulæ, and the full index at the end is a valuable feature. As a work it is a standard in the German language and it deserves the same position in the English. The translation is satisfactory, and the work of the publishers is neat and attractive.

